# The burden of community-acquired pneumonia in the elderly: the Spanish EVAN-65 Study

**DOI:** 10.1186/1471-2458-8-222

**Published:** 2008-06-27

**Authors:** Olga Ochoa-Gondar, Angel Vila-Córcoles, Cinta de Diego, Victoria Arija, Monica Maxenchs, Montserrat Grive, Enrique Martin, Josep L Pinyol, EVAN-65 Study Group

**Affiliations:** 1 Primary Care Service of Tarragona-Valls, Institut Català de la Salut, Tarragona, Spain; 2 Department of Public Health, Primary Care Service Camp de Tarragona, Institut Català de la Salut, Tarragona, Spain; 3 The EVAN-65 Study Group

## Abstract

**Background:**

Community-acquired pneumonia (CAP) is generally considered a major cause of morbidity and mortality in the elderly. However, population-based data are very limited and its overall burden is unclear. This study assessed incidence and mortality from CAP among Spanish community-dwelling elderly.

**Methods:**

Prospective cohort study that included 11,240 individuals aged 65 years or older, who were followed from January 2002 until April 2005. Primary endpoints were all-cause CAP (hospitalised and outpatient) and 30-day mortality after the diagnosis. All cases were radiographically proved and validated by checking clinical records.

**Results:**

Incidence rate of overall CAP was 14 cases per 1,000 person-year (95% confidence interval: 12.7 to 15.3). Incidence increased dramatically by age (9.9 in people 65–74 years vs 29.4 in people 85 years or older), and it was almost double in men than in women (19.3 vs 10.1). Hospitalisation rate was 75.1%, with a mean length-stay of 10.4 days. Overall 30-days case-fatality rate was 13% (15% in hospitalised and 2% in outpatient cases).

**Conclusion:**

CAP remains as a major health problem in older adults. Incidence rates in this study are comparable with rates described in Northern Europe and America, but they largely doubled prior rates reported in other Southern European regions.

## Background

Community-acquired pneumonia (CAP) is a relatively frequent infectious illness which causes important morbidity worldwide [1,2] The reported incidence rates of radiographically confirmed CAP in different populations have varied between 1.3 and 11.6 cases per 1,000 inhabitant-year, [3-12] with the highest rates in older adults [13-15].

Nowadays, CAP cases in older adults increase as a consequence of an overall increase in the elderly population (persons aged 65 years or older) [16] In developed countries, almost one half of the total hospitalisations for pneumonia occur in patients over 65 years and pneumonia is a leading cause of death among this age group [17-21]. However, despite the recognised importance of CAP in the elderly, information on the epidemiology of CAP in this age group is limited and the true burden of the disease is not well known, considering that incidence and mortality rates in elderly populations have largely varied in different studies.

Incidence rates varying between 2–40 cases per 1,000 elderly person-year and case-fatality rates between 7–35% have been reported for elderly patients in Europe and North America during the last two decades [3-5,7-15,20-23]. However, most prior studies were conducted among general adult populations and included only a limited number of elderly individuals,[3,7,18,10-12] many studies focused on hospitalised patients,[4,9,15,20-23] and few studies focused on the possible contribution of outpatient cases [14,24-26] To our knowledge, only two population-based studies specifically focused on older adults, including hospitalised and outpatient cases of CAP, have been published [13,14].

In this study, we have assessed the overall burden of CAP (hospitalised and outpatient cases) in a population-based cohort of Spanish community-dwelling elderly individuals followed between 2002 and 2005. In prior reports we assessed the effectiveness of the pneumococcal vaccine in preventing pneumonia and death in this same cohort [27,28].

## Methods

### Design, setting and study population

We conducted a population-based prospective cohort study including all community-dwelling individuals 65 years or older assigned to 8 Primary Health Care Centres (PHCC) in the region of Tarragona, a mixed residential-industrial urban region on the Mediterranean coast in Catalonia, Spain. Cohort members were followed from when the study started (January 1, 2002) until the occurrence of the first event, the enrolment from the PHCC ceased, death, or until the end of the study (April 30, 2005). Mean temperatures in the study area for summer and winter seasons through the study period were 23.2°C (73.8°F) and 9.8°C (49.6°F), respectively.

In the Spanish Health Care System, the same as in the study area, all persons are assigned to a PHCC, where the General Practitioner files relevant medical details on patients during primary care visits, and there are different reference Hospitals according to geographical and demographic data. When the study started, the Health District of Tarragona had 12 PHCCs with an overall assigned population of 134,232 all-age inhabitants. The selection of the 8 participating PHCCs was not randomised and they were chosen taking into account the existence of electronic clinical registries working since 1998 or before. The other 4 PHCCs in the Health District were not included because they had only computerised clinical records more recently. The study cohort included all community-dwelling individuals assigned to the eight participating PHCCs, who were 65 years or older at the start of the study (an amount of 11,240 individuals with a mean age of 74.6 [SD: 7.5] years-old at baseline) The main characteristics of the study population are extensively described elsewhere [27,28] The study was approved by the ethical committee of the Catalan Health Institute and was conducted in accordance with the general principles for observational studies set out by this institution.

### Sources of data

All participating PHCCs had an institutional database with registries of administrative data, medical conditions and diagnoses associated with outpatient visits coded according to the International Classification of Diseases, 9^th ^Revision, Clinical modification (ICD-9). This institutional database and the primary care clinical records of each cohort member were respectively used to identify and validate cases of outpatient CAP which occurred among cohort members during the survey. An active surveillance program on CAP was established before the study period and primary care physicians were asked to register all cases of CAP radiographically confirmed in their patients.

The hospital discharge diagnoses database and the clinical medical records of the three participating reference hospitals (Joan XXIII, Santa Tecla, and Pius Hospital) were used to identify hospitalisation for CAP in cohort members during the study period.

### Outcome measure and definitions

Primary endpoints were CAP and death from CAP. Pneumonia was defined when a new radiological infiltrate was identified with one major criteria (cough, expectoration, and fever) or two minor criteria (dyspnea, pleuritic pain, altered mental status, pulmonary consolidation on auscultation, and leukocytosis) [22]. Death from pneumonia was considered when the patient died (in-hospital or not) within the first 30 days after the diagnosis [29,30].

Hospitalisations for CAP were identified on the basis of the first-listed code in the Hospital discharge database (ICD-9 codes for pneumonia: 480 to 487.0), whereas outpatient CAPs were primary care or emergency visits with an ICD-9 code registered for pneumonia in the PHCCs databases. All the cases of CAP (hospitalised and outpatient) were radiographically confirmed and validated by checking clinical records.

Given that PHCCs and Hospital medical records of case patients were reviewed several weeks after the diagnosis of CAP, the physician reviewer verified that x-ray findings improved with treatment and excluded the possibility that the episode was not a readmission (defined as a re-hospitalisation within 30 days after inpatient treatment of CAP) or a nosocomial pneumonia (defined as a pneumonia acquired after hospital admission at any time) [31,32].

### Statistical analysis

Incidence rates of CAP were calculated as person-year, considering that in the denominator the total persons-time for the study period was simply the sum of the person-time contributed to each individual during the study period. Case-fatality rates were calculated by dividing the number of cases of deaths from CAP by the absolute number of CAP cases in each age- and sex- specific stratum. Event rates were based on the first episode of CAP occurring during the study period and they do not include multiple events per person. Confidence intervals (CI) were used to compare incidence and mortality rates between the different categories and population groups. Chi-squared and Fisher's tests were used to calculate p-values in the comparison of proportions, whereas Student's test and one-way analysis of variance were used to compare continuous variables. Statistical significance was set at p < 0.05. The analyses were performed using Stata/SE version 9.1 (Stata Corp.).

## Results

The 11,240 cohort members were observed for a total of 33,905 person-year. Overall, 43.5% of the subjects were male, 55.2% were aged 65–74 years and 10.5% were aged 85 years or older at baseline. Of the 11,240 cohort members, 1,497 (13.3%) died during the 40-month study period and 315 (2.8%) were lost patients during follow-up. Table 1 shows study population and person-time followed according to sex and age-groups.

**Table 1:** Study population and person-year followed according sex and age groups^a^.

**Sex**	**Number of persons (No. of person-year followed)**		
	
**Age group**	**Male**	**Female**	**Overall**
**65–74 yrs**	2897 (9016)	3308 (10573)	6205 (19589)
**75–84 yrs**	1578 (4533)	2281 (6887)	3859 (11420)
**85 yrs or more**	417 (1001)	759 (1895)	1176 (2896)
**Overall age groups**	4892 (14550)	6348 (19355)	11240 (33905)

^a^Age was considered when study started on January 1, 2002.

A total of 380 cohort members who had a presumptive episode of hospitalisation for CAP were identified on the basis of the hospital discharge databases. Information on 369 (97%) of these events was available for clinical record review, of which 355 were validated as CAP cases and 14 were excluded (nosocomial pneumonia or other diagnoses). Furthermore, a total of 132 individuals with an episode of presumptive outpatient pneumonia were identified according to ICD-9 pneumonia codes from emergency or ambulatory visits, but only 118 cases (89%) were included as radiologically-confirmed outpatient CAP after review of the clinical records. This means that annual incidence rates (per 1,000 elderly person-year) was 13.95 for overall CAP (95% CI: 12.72 to 15.31), whereas incidences were 10.47 for hospitalised CAP (95% CI: 9.42 to 11.61) and 3.48 for outpatient CAP (95% CI: 2.88 to 4.17).

Incidence rates of overall CAP increased significantly by age groups (9.9 in 65–74 years, 16.9 in 75–84 years and 29.4 in people 85 years or more; p < 0.001) and were almost two-fold higher in males than in females (19.3 vs 10.1; p = 0.001).

Table 2 shows incidence rates of hospitalised and outpatient CAP, according to sex and age groups. The incidence rate was consistently higher among men than among women, across all age groups and for both hospitalised and outpatient CAP, although the difference was not always statistically significant for outpatient CAP.

**Table 2:** Incidence Rates (IR) of community-acquired pneumonia (CAP), by age group and sex, during the study period.

**Sex**	**Male**		**Female**		
	
**Age group**	**No. CAP**	**IR**^a^**(95% CI)**^b^	**No. CAP**	**IR**^a^**(95% CI)**^b^	**P-value**^c^
**65–74 yrs**					
**Hospitalised CAP**	100	11.09 (9.04–13.47)	45	4.26 (3.11–5.69)	0.000
**Outpatient CAP**	27	2.99 (1.98–4.35)	23	2.18 (1.38–3.26)	0.257
**All CAP**	127	14.09 (11.76–16.73)	68	6.43 (5.00–8.14)	0.000

**75–84 yrs**					0.000
**Hospitalised**	90	19.85 (16.00–24.34)	60	8.71 (6.66–11.19)	0.030
**Outpatient**	24	5.29 (3.40–7.86)	19	2.76 (1.56–4.30)	0.000
**All CAP**	114	25.15 (20.80–30.13)	79	11.47 (9.10–14.27)	

**85 yrs or more**					
**Hospitalised**	29	28.97 (19.50–41.32)	31	16.36 (11.15–23.13)	0.234
**Outpatient**	10	9.99 (4.80–18.30)	15	7.92 (4.44–13.02)	0.575
**All CAP**	39	38.96 (27.86–52.85)	46	24.27 (17.83–32.23)	0.026

**Overall age groups**					
**Hospitalised**	219	15.05 (13.14–17.16)	136	7.03 (5.90–8.30)	0.000
**Outpatient**	61	4.19 (3.21–5.38)	57	2.94 (2.23–3.81)	0.054
**All CAP**	280	19.24 (17.08–21.60)	193	9.97 (8.62–11.47)	0.000

^a^Incidence Rates are estimated for 1,000 person-year.

^b^CI denotes confidence interval

^c^P-values were calculated using Chi-squared test.

Overall, 75.1% of CAP episodes were hospitalised and 24.9% were managed as outpatients. Percentages of CAP cases requiring hospitalisation were 74.3% among patients 65–74 years, 77.7% in 75–84 years and 70.4% in 85 years or older (p = 0.429).

The mean days of hospitalisation was 10.4 days (SD: 8.02). We observed that the mean length-stay was slightly higher in men than in women (10.8 vs 9.8; p = 0.241). According to age groups, the means of length-stay were 11 days in people 65–74 years, 10.3 days in people 75–84 years, and 9.2 days in people 85 years or older (p = 0.332).

Twenty (5.6%) of 355 hospitalised CAP were admitted in the Intensive Care Unit (ICU), with a mean stay of 10.6 days (SD: 9.9).

**Figure 1:**
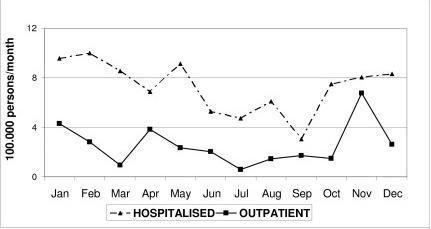
Incidence of CAP in elderly people, according to monthly distribution of hospitalised and outpatient cases, in the region of Tarragona (Spain) between January 2002 and April 2005.

The mean incidence of CAP was more than two-fold higher in winter than in summer (9.1 vs 4.1 cases per 100,000 persons-week, respectively). Figure 1 shows the monthly distribution of incidence rates for both hospitalised and outpatient CAP during the study period.

As for 30-days mortality after the diagnosis, there were 60 deaths from CAP (two deaths among CAP cases managed as outpatient, three deaths of patients in Emergency Unit before hospitalisation, and 55 deaths among hospitalised patients). This means that the annual mortality rate from CAP was 177 per 100,000 elderly persons-year (95% CI: 135 to 228). Overall, 30-days case-fatality rate was 12.7%. Mortality was only 2% for CAP cases managed as outpatient, whereas it reached 15% for CAP requiring hospitalisation (40% for patients admitted in the ICU). Case-fatality rate did not differ significantly between sex (13.9% in male vs 10.9% in female, p = 0.328), but increased dramatically with increasing age (7.2% in people 65–74 years, 13.5% in people 75–84 years and 23.5% in people 85 years or older; p = 0.002). Table 3 shows the different CAP case-fatality rates according to sex and age strata.

**Table 3:** Overall 30-days mortality from community-acquired pneumonia (CAP) in elderly subjects, according to age and sex strata.

**Sex**	**Mortality within 30-days after diagnosis of community-acquired pneumonia**				
	
	**Male**		**Female**		
	
**Age groups**	**No. of events Deaths/CAPs**	**Case-fatality % (95% CI)**^a^	**No. of events Deaths/CAPs**	**Case-fatality % (95% CI)**^a^	**P-value**^b^
**65–74 yrs**	11/127	8.7 (4.4–14.5)	3/68	4.4 (0.9–12.4)	0.386
**75–84 yrs**	19/114	16.7 (10.3–24.8)	7/79	8.9 (3.6–17.4)	0.118
**85 yrs or more**	9/39	23.1 (11.1–39.3)	11/46	23.9 (12.6–38.8)	0.927
**Overall people**	39/280	13.9 (10.1–18.5)	21/193	10.9 (6.9–16.2)	0.328

^a^CI denotes confidence interval

^b^P-values were calculated, where appropriate, by Chi-squared or Fisher's tests.

## Discussion

In the present study, we have assessed population-based incidences and 30-days mortality of CAP among people 65 years or older in a well defined geographic area on the Mediterranean Coast of Spain during a consecutive 40-month study period. To our knowledge, this is the first contemporary study to assess rates of both hospitalised and outpatient CAP specifically focused on community-dwelling elderly people in a European country.

We have found that the incidence of CAP in our population (14 episodes per 1,000 elderly person-year) is more than three-folds higher than incidences previously reported in other Mediterranean regions[8,10-12] and it is more similar to CAP incidences reported in Northern Europe or North America for elderly population groups [7,9,14,15].

In Europe, in those population-based studies that evaluated the incidence of CAP in the general adult population, the reported incidence rates of CAP among the subgroup of people aged 65 years or older varied widely from 2 cases per 1,000 elderly person-year in Spanish individuals[10] to 24 cases per 1,000 reported among Finnish elderly people [7]. In three different regions along the Spanish Mediterranean Coast, the reported incidences of overall CAP have ranged from 1.2 to 1.8 cases per 1,000 all-age adult population (2.3 to 3.8 cases per 1,000 among people 65 years or older) [8,10,11] Incidence rates of 1.7 and 3.3 episodes of CAP per 1,000 person-year have been recently reported among Italian all-age adults and elderly individuals, respectively [12].

If we compare our results with those recently reported by Jackson et al[14] in a population-based cohort study that evaluated the incidence of CAP among 46,237 elderly individuals in Washington State between 1998–2001, we can observe similar incidence rates of CAP requiring hospitalisation (10.5 per 1,000 in this study vs 11.5 per 1,000 in the Jackson's report). In contrast, the incidence of outpatient CAP was largely higher in USA elderly than in the present study (16.8 vs 3.5 cases per 1,000 person-year), which probably reflects differences in the definition criteria and management of outpatient CAP in both studies. In the present study (excluding three CAP cases who died in the emergency room before their possible hospitalisation), 15.5% of hospitalised CAP and 1.7% of outpatient CAP died within 30 days after the diagnosis, while in Jackson's report these percentages were 12.5% and 0.4% respectively. Overall, 4% of all the deaths in the cohort during the study period occurred within 30 days after a CAP diagnosis in this study versus 3.6% in Jackson's report.

In this study, incidence of CAP increased dramatically with ageing, achieving the highest rate in people aged 85 years or more, where 29 cases per 1,000 person-year were observed. According to sex and age strata, very elderly men are at the greatest risk considering that one episode of CAP can be expected every year for every 25 men aged 85 years or older. Similar trends in the incidence rates stratified by age have been reported in most prior epidemiological studies, considering that the frequent association between increasing age and presence of underlying diseases accounts for an increased morbid-mortality due to CAP in the oldest adults [17,18,21]. It must also be noted that in this study the 30-days case-fatality rate was three fold higher among patients 85 years or older than in patients 65–74 years, which supports the important specific role of age as a predictor of 30-days mortality among patients with CAP, as the pneumonia severity score reflects [29].

In the present study, the high rate of hospital admission may be explained by the characteristics of the study cohort (mean age: 75 years) and the characteristics of the "Tarragona region" where the study was conducted, particularly in relation to the easy accessibility to the reference hospital so that many patients sought medical care directly from the emergency service of the hospital rather than visiting a primary care physician. Interestingly, although the proportion of hospitalised cases was high in the present study, the incidence rate of CAP requiring hospitalisation resembles the rates reported in population-based studies conducted in the USA, where figures between 10.1 and 11.5 per 1,000 elderly person-year have been reported [9,14].

## Conclusion

Our study has several strengths. Study design was population-based, outcome measures and definitions were based on defined criteria in classical studies and meta-analysis[30] and all cases of CAP were radiographically confirmed and validated by clinical record review. However, considering that case finding was primarily restricted to hospitalisations or outpatient visits recorded with an ICD-9 code 480–487 and chest-radiograph was needed to validate each case, one limitation of this study could be the possible under-identification of CAP events. This possible problem is likely to be more important for CAP treated on an outpatient basis than in hospitalised cases. For outpatient CAP, although our study design allowed for the inclusion of all cases of pneumonia diagnosed by primary care physicians in the study area, it is possible that some patients with mild symptoms were missed because they were not referred to the hospital or the emergency unit for evaluation, because a chest radiograph was not ordered, or because pneumonia diagnosis code was not recorded in the primary care clinical record. A second limitation of this study lies in the fact that, although it includes all community dwelling elderly persons assigned to 8 different PHCCs, overall study population includes only persons living in a single geographic area, and it may not be possible to extrapolate the findings to the Spanish population as a whole.

In the next few years, population-based studies focused on the incidence and epidemiology of CAP are needed to investigate the true burden of CAP at the beginning of the third millennium in different settings and study populations. Specifically, considering elderly people, the burden of CAP is highest in these subjects and the effects of currently implemented preventive measures such as smoking cessation or pneumococcal vaccination (largely focused in this high-risk group) should be evaluated on the basis of actual surveillance and incidence data.

## Competing interests

The authors declare that they have no competing interests.

This study was supported by a grants from the "Fondo de Investigación Sanitaria" of the Spanish Health Ministry (expedients FIS PI-021117 and PI-050231) and Jordi Gol i Gurina Foundation.

## Authors' contributions

OO-G and AV-C designed the study, assessed outcomes, and wrote and edited the paper; AV-C coordinated the study; CdeD, MM, MG and EM obtained the data; VA and JLP did the statistical analysis.

## Appendix

The following persons are EVAN-65 Study Group members:

A. Vila-Córcoles, X. Ansa, N. Saún, A. Gómez, J. Fort, M. Piqueras, J. Grifoll, JL. Pinyol, J. Bladé, D. Montanyes, J. Daniel (Primary Care Service of Tarragona-Valls); N. Sarrá, JM. Roca, M. Grivé, R. Antón (Primary Care Center of Bonavista-La Canonja); J. Boj, B. Rull, CM. Fuentes, E. Satué, MJ. Solís, MC. de Diego, B. Fernández, V. Silvestre, MA. Puig, X. Bria (Primary Care Center of Torreforta-La Granja); I. Noguera, O. Ochoa-Gondar, M. Herreros, F. Grifoll (Primary Care Center of Sant Pere i Sant Pau); C. Llor, F. Bobé, M. Maxenchs, M Perez-Bauer (Primary Care Center of Tarraco); J. Balsells, E. Martín, L. Clotas, A. Serrano (Primary Care Center of Sant Salvador); L. Palacios, F. Gallego, C. Ferrández, E. Salsench (Primary Care Center of Salou); F. Ester, S. Montserrat, G. Cando (Primary Care Center of Morell); M. Alvarez, I. Hospital, I. Guinea, MM. Juarez, C. Bayona, D. Llovet (Primary Care Center of Valls); A. Vilanova, F. Gómez-Bertomeu, JM. Santamaria, A. García-Fuertes (Hospital Joan XXIII, Tarragona); X. Raga, X. Clivillé, J Bitria, MC. Daufí (Hospital Santa Tecla, Tarragona); T. Benet, JM Villó (Pius Hospital, Valls).

## Pre-publication history

The pre-publication history for this paper can be accessed here:


